# An Account of an Epidemic Bilious Fever, Which Prevailed in Indiana in 1845

**Published:** 1846-08

**Authors:** Thomas W. Fry

**Affiliations:** Crawfordsville, Indiana


### THE WESTERN JOURNAL OF MEDICINE AND SURGERY.

AUGUST, 1846.

**Art. I.**—*An Account of an Epidemic Bilious Fever, which prevailed in Indiana in* 1845. By Thomas W. Fry, M.D., of Crawfordsville, Indiana.

In the summer and fall of 1845, this section of Indiana was visited by an epidemic almost unparalleled in its medical history. It was not confined to any particular localities; but its victims were found sick upon the hills and in the valleys, upon the bluffs of water courses, and the margins of marshy ponds, upon the high, dry, rolling timber lands, and in the vicinity of the stagnant pools of the barrens and prairies. In the early part of the summer there were some severe cases of cholera morbus, and many cases of a mild intermittent fever. Towards the latter part of July remittent and congestive fevers appeared in our midst, and almost the entire population was suddenly stricken with disease.

The object of the present article is to give a brief account of Remittent Fever, the form in which the disease prevailed most extensively. The description must necessarily be imperfect, since the privilege of post-mortem examinations could not be had.

Although the very existence of remittent fever has been denied, it is now known to possess certain characteristics distinguishing it from all other fevers. "The term is employed to designate a fever of which the symptoms undergo at intervals during its course a marked abatement or diminution, which is called a remission;" the paroxysms of fever, followed by remissions, generally occurring daily. The type of the disease of which I am about giving a history, was variant; in some cases manifesting a decided tendency to the intermittent form; in others, running into continued fever. The remissions, at times, were so distinct that they were with difficulty distinguished from intermissions; and again they were so slight, and of so short duration, as to be scarcely recognisable. Intermittent and remittent fevers were, by Cullen, identified. Mackintosh remarks, "that remittent fever is a disease of warm climates, and when the skin is yellow has obtained the name of yellow fever. It may be regarded more properly as forming the mean degree in the scale of periodic or marsh fevers, of which intermittent and yellow fever constitute the extreme points. A more intense operation of the febrific cause than is required for the production of intermittent fever engenders remittent, and the more violent the latter the more remote is its character from intermittent: or in other words, the less perceptible the remissions." This may be inferred from the fact that in countries where this disease prevails, strangers suffer more intensely from it than permanent residents.

The treatment which I pursued having been somewhat at variance with the usual plan recommended by systematic writers, I shall give a detailed history of a few cases, prefacing them with a brief enumeration of the symptoms preceding and accompanying the attack. As a general thing, there was some previous indisposition, such as headache, languor, loss of appetite, and debility. The tongue was coated in some cases with a white or yellowish white, and, in others, with a brown fur, accompanied with bitter taste, and considerable thirst; occasionally the tongue was red in the centre, and furred on either side. A sense of weight was generally felt in the epigastrium, and tenderness on pressure in that region and in the left hypochondrium. The spleen, in many cases, was evidently enlarged, and may have been instrumental in the production of pain. The pain, however, was never so great as to demand bloodletting; the application of mustard plasters generally afforded relief. The pulse was weak, frequent, compressible, and sometimes irregular; varying very little in frequency, during the remissions, from the natural standard; seldom hard, strong, or bounding, even during exacerbations. Vomiting frequently occurred in the early stages of the disease, yellow, an1 sometimes, thin green matter being discharged. The urine, in most cases, was scanty and high colored, producing a burning sensation as it passed.

The attack, in almost every instance, was ushered in by a sense of chilliness, generally recurring at intervals of forty-eight hours, evidently showing the type to be tertian. There was, however, some variation in this respect; occasionally there were diurnal exacerbations and remissions, the exacerbations being much more severe on alternate days. The mind was generally clear, sometimes confused, with occasional bursts of delirium. In a few cases there was "the striking peculiarity noticed first by Robert Jackson, M.D., in 1817," and subsequently by Joseph Brown, M.D., in the Encyclopædia of Practical Medicine, "in which the mind retains its acuteness of perception and vigor of reasoning, but there is one erroneous impression so firmly fixed that no argument can shake it." In one case the individual imagined his head and limbs to be separated from his body and occupying different localities; he was afraid to take food or medicine lest his stomach should not be in the proper position for its reception. Many were deeply impressed with the idea that the disease must necessarily terminate fatally. This impression operated powerfully in retarding the progress of recovery, but was not often realized. The vast amount of sickness threw a gloom over the whole community, which produced desponding impressions on the minds of all patients.

*Cause.*—The conflicting opinions on this subject would indicate that the cause of intermittent, remittent, and continued fever is still shrouded in mystery. The generally received opinion, that malaria is the cause of fever, has been controverted in an able article on the Pathology of Fever by Robert Willis, M.D. It is remarked by Dr. Willis that "the problem of the highest interest in medical science that waits for solution at the present moment is, perhaps, this:—To explain the cause of the unhealthiness of so many tropical climates, and of alluvial and marshy countries generally, where the summer temperature is high." He continues, "that after a careful study of the subject, he entirely concurs in opinion with those who, from their experience in tropical climates have felt themselves authorised to deny the existence of any specific contagion or miasm, the product of vegetable matter in a state of decay under the combined influence of heat and moisture. As chemists we are perfectly familiar with the products of the decomposition of vegetable substances under such circumstances, and as physiologists with the effect of gases then evolved upon the animal economy, which certainly bears no resemblance to the phenomena of remittent and intermittent fever. According to my ideas, marsh miasm and malaria are nothing more nor less than moist warm air—air, excessively moist, considered in connexion with its own temperature, and the temperature of the human body." In support of this opinion he alludes to the experiments of M. Delaroche, which go to prove that animals confined in stove rooms experience more speedy and fatal effects when the air is moist and of a high temperature than they do when the air is hot and dry. Death, however, is the result in both instances, but is produced in different ways;" in the former they die as they do when covered with an impervious glaze; "the conditions necessary for the excess of oxidised plasma, and the removal of deoxidised plasma are wanting, and life ceases as a matter of course." He also alludes to the unhealthy regions on the western coast of Africa, where the mean temperature is about 80° Fah., and the atmosphere close upon the point of saturation with humidity; here man is on the verge of circumstances that are even incompatible with existence. The sudoriparous glands, he contends, present a larger quantity of watery vapor than can be taken up by the surrounding atmosphere, with sufficient rapidity to meet the wants of the system in its state of excitement; fever ensues, and life is almost of necessity the forfeit. Those mysterious powers spoken of by tropical writers as solar and lunar influences are, according to Dr. Willis, nothing more than effects of exposure to the sun's heat and the increased moisture of the air which follows in the temperature of the night season. This heat, aided by moisture, produces functional derangement of the skin, which prevents the free access of the oxygenated plasma so necessary to the existence of life.

To this forcible and ingenious article of Dr. Willis it is replied by Dr. McWilliams, late senior medical officer to the Niger Expedition: "That heat and moisture are conditions of the atmosphere which readily admit of quantitative determination, by methods in common use; and if fever were caused by them alone in Europeans in the tropics, it should prevail whenever their amount is the same. Now by reference to the meteorological tables in my work, the temperature and dew-point outside the niger, where no fever occurred, and while in the river, were as follows:

| | Tem., 3 p. m. | Dew-point, 3 p. m. |
|---|---|---|
| Passage from Sierra Leone to Arca | 87·77 | 70·61 |
| Outside Niger from 9th to 12th August | 70·00 | 73·70 |
| In the river and ascending to Abek | 80·00 | 74·00 |
| At Abek, Idduk, and the confluence of the Niger, and Tchadda, to Sept. 21st | 84·60 | 73·50 |
| Confluence of the Niger and Tchadda to Egga | 86·60 | 72·00 |

Thus, though the expedition was exposed from the first of July to the beginning of August, to air containing more moisture, and but little inferior in temperature, at the hottest part of the day, to any experienced within the river, not a case of fever made its appearance until the 4th of September, three weeks after it entered the river, and had been exposed to the emanations from the ordinarily recognised sources of malaria. Similar results have been observed elsewhere; in Barbadoes, for instance, no fever occurred among the troops in the garrison during August, September, or October, 1841, and although in November a very violent fever broke out, the temperature of the air was lower than in August, and the dew-point lower than in September. Their means were as follows:

| | Temperature, 3 p.m. | Dew-point, 3 p.m. |
|---|---|---|
| August, - - - - - | 83·77 | 70·61 |
| September, - - - | 82·13 | 73·78 |
| October, - - - - - | 82·31 | 72·67 |
| November, - - - | 82·83 | 71·67 |

"Hence, he concludes, the connexion between heat and humidity of the atmosphere and severe remittent and yellow fever is by no means so clear as Dr. Willis would have us suppose." Many other facts are related by Dr. McWilliams in relation to the West coast of Africa in direct opposition to those of Dr. Willis.

As to the cause of the general prevalence of the disease in this region of country my mind is not fully satisfied. In many parts of it there exist most of the ordinarily recognised sources of malaria; in many others none at all, or if any, to a very limited extent. One fact, however, I observed, which may have had some agency in generating the specific poison. There were frequent showers of rain, followed by a very hot sun, which produced an unusually rapid decay of vegetable matter. This was observed more particulary in the latter part of the season when the leaves of the trees were beginning to fall.

The mean temperature of those months in which the sickness prevailed most extensively, was as follows:

| | |
|---|---|
| July, - - - - - - | 76·84 |
| August, - - - - - | 76·86 |
| September, - - - - - | 67·18 |
| October, - - - - - | 55·96 |
| November, - - - - - | 43·43* |

In July the thermometer ranged, at 12 o'clock, throughout the whole month, from 70·67 to 94·00, never falling below or rising above these points.; in August its range was from 70·00 to 91·00; in September from 55·00 to 86·00. By far the greater number of cases occurred in August and September, months in which dews are generally heavy. The attacks generally followed exhausting fatigue, exposure to a hot sun, or the damp night air. What agency malaria had in producing the disease I could not determine, since it prevailed as extensively in those sections of country esteemed healthy, as it did in those that were considered malarious.

Case I.—Mrs. Vanarsdale 45 years of age, a woman of uncommon health and strong constitution, was taken 13th July with severe headache, pains in the loins and lower part of the back, a general feeling of lassitude, dizziness on rising, slight

---

* Taken from the Meteorological Tables of Rev. William Twining, Professor of Mathematics in Wabash College.

nausea and oppression about the epigastrium, intelligence good. Gave an emetic of ipecacuanha, which relieved the stomach of a large quantity of bilious matter; calomel and rhubarb of each grs.viii, to be taken at bed time.

14th. The medicine produced several stools; the patient passed a restless night; skin hot and dry; tongue coated with a brown fur; thirst great; giddiness; considerable prostration; bitter taste; no tenderness in the epigastrium, but a severe sense of weight there; severe pain in the head, but no delirium; anxious expression of countenance; pulse weak and frequent. Calomel and Dover's powder of each grs. iii., to be taken at intervals of three hours, through the day, followed by a small dose of castor oil in the evening.

15th. Passed a more comfortable night; intelligence good; slight moisture on the skin through the night; mouth still dry; no appetite; some thirst; headache; eyes dull and slightly tinged with yellow; stools green and liquid; considerable exacerbation towards evening, with tenderness in the epigastrium, which is relieved by the application of mustard; stools still liquid and green. Two powders as on yesterday; spts. nitre with a small addition of ipecacuanha every two hours through the day.

16th. Headache much less severe; intellect perfectly clear; no nausea; mouth clammy, not so dry; free perspiration through the night; stools consistent; no return of pain in the epigastrium; pulse frequent and compressible. The condition of the patient seeming to demand quinine, a small portion was given, but as it was not borne well, was omitted, and the nitre and ipecac. continued. Blue pill and rhubarb at night.

17th. General condition much improved; remission more distinct; still very little appetite. Quinine grs. ii. every two hours, till ten grains have been taken.

18th. Slept well; skin moist; tongue cleaning off; appetite a little improved; urine, which up to the present time had been scanty and high colored, is clear and more abundant; stools less frequent and more consistent. Quinine to be continued as yesterday. The patient, though apparently free from disease, remained weak for several days, when she was attacked with diarrhœa, followed by severe paroxysms of fever. These symptoms were relieved by a similar course of treatment, in about the same length of time.

Case II.—Mrs. Walton, 38 years of age, had enjoyed but poor health for some months, and for several days previous to the time when I first saw her, had suffered from severe headache and nausea, accompanied by occasional vomiting of bilious matter; a sense of weight in the epigastrium, and pain in the left hypochondrium, and pains in the back and loins. On the morning of the 13th August I found her laboring under the above enumerated symptoms. Skin hot and dry; tongue thickly coated with yellowish white fur; excessively bitter taste; extremities cool; chilly sensations running up the back; the mind slightly disturbed; bowels bound; pulse moderately full and frequent. An emetic of ipecac. was given, by the operation of which a large quantity of bile was discharged. Blue pill at night, to be followed by castor oil in six hours.

14th. The condition of the patient is more comfortable than it has been for several days, but the sense of weight and tenderness is not entirely removed; pulse is feeble and frequent; tongue thickly coated; great oppression; several scanty, liquid stools; slight nausea; skin dry; some thirst; conjunctiva yellow; urine still scanty and high colored; some chilly sensations in the forenoon; mind in a better condition, but when interrogated the patient weeps. Spirits nitre and paregoric to be given through the day; blue pill and rhubarb at night; mustard to be applied to the extremities and epigastrium.

15th. Pulse and urine as yesterday; stools less frequent and more consistent; pain in the head continues about the same; slept but little; the face and upper part of the body a little moist; considerable prostration. Quinine gr. i. ordered to be given every hour, but could not be taken in consequence of increased febrile excitement. Spirits nitre and ipecac. substituted; another dose of blue mass and rhubarb at night.

16th. The paroxysm of fever passed off with copious perspiration; tongue still coated with a white fur; cephalalgia very slight; no epigastric tenderness; pulse moderately full and soft; urine more copious and more of a straw color; no appetite; some thirst; although there is a general modification of the symptoms, the mind is fixed upon the necessary fatality of the disease; mouth clammy; stools more frequent. Blue pill grs. iv, rhubarb grs. iv, every three hours till there is free discharge from the bowels.

17th. Three doses of blue mass and rhubarb acted well upon the bowels, after which there was some refreshing sleep; tongue still furred; skin soft; thirst diminished; desires a little chicken broth; discharges from the bowels becoming too frequent; Dover's powder grs vi checks them; quinine grs. ii every hour for six hours.

18th. Strength slightly increased; passed a tolerably comfortable night; thirst moderated; appetite some better; tongue cleaning off; countenance more cheerful. Chicken broth for diet, and quinine grs. v to be taken in the course of the day.

19th. Slight exacerbation yesterday evening, but less than on any preceding day; intelligence perfectly good; no cephalalgia; pulse approaching a natural standard; she continues weak; wine and mild diet recommended. Visits discontinued. Patient improved for several days, when she was seized with severe pains in the bowels, followed by frequent and slight discharges of bloody mucus, but these symptoms were soon relieved by the ordinary remedies.

Case III.—Mrs. Quick, 34 years of age, very much worn down by the fatigues of nursing a sick family, was taken on the 28th August with a slight chill, followed by a severe paroxysm of fever, cephalalgia, delirium, and great restlessness. The attack was preceded by several days of languor and general indisposition. The sense of weight and tenderness at the epigastrium and hypochondrium, present in all the cases to which I was called, was rather greater in hers than usual; considerable prostration and a tendency to stupor also marked her case. Ipecacuanha was given in sufficient quantity to excite free vomiting, and an unusually large quantity of bilious matter was thrown up. Calomel and rhubarb of each grs. viii, to be taken at night.

29th. The powder taken acted freely on the bowels, carrying off much dark, offensive matter, greatly to the relief of the head and back; pulse weak and frequent; tongue coated with a brown fur; mouth dry; much thirst; sense of weight and tenderness diminished; urine scanty and high colored; mind slightly wandering; skin dry, but not so hot as yesterday. Spirits nitre and ipecac. to be given every two hours through the day; a dose of aperient pills at night.

30th. No distinct chill yesterday; an exacerbation in the afternoon, which went off with excessive perspiration; rested badly; drowsy this morning; tongue still coated and clammy; urine more freely discharged; no delirium, but considerable restlessness; giddiness upon rising; no appetite; thirst. Prescription as yesterday; blue pill in place of the aperient.

31st. Chill came on yesterday about 11 o'clock, lasted for several hours, and was followed by high fever, delirium, epigastric tenderness, and cephalalgia; had passed a restless night; slight remission this morning. Mustard applied to the extremities and epigastrium; the nitre continued, and rhubarb and jalap given in sufficient quantities to act freely on the bowels.

Sept. 1st. Paroxysm of fever yesterday evening much less violent than that of the preceding day. Bowels were freely opened; skin softer, with some moisture on the face and upper part of the body; mouth less clammy; conjunctiva tinged with yellow; countenance expressive of anxiety; intelligence good; no appetite; no cephalalgia. The remission was so distinct as to justify the exhibition of quinine, which was given in two grain doses every hour for five hours. Blue pill and rhubarb at night.

2d. Considerable improvement from yesterday. Patient rested well through the night; perspiration copious to-day; no headache; tongue cleaning off; some appetite; slight improvement in strength; no thirst; intellect perfectly clear. Quinine continued.

The patient continued feeble for several weeks, occasionally suffering from slight paroxysms of fever, which were finally relieved by the use of quinine.

Two cases committed to my care terminated fatally, but as post-mortem examinations were not permitted, it is unnecessary to write them out in detail. One of them was complicated with thoracic disease.

*The General Treatment of Fever.—Bleeding.* So far as my knowledge extends, bleeding is recommended by all systematic writers, with few exceptions; some, it is true, oppose the use of the lancet upon the ground that the blood is in what is considered a dissolved state. The conviction of the impropriety of bloodletting in the disease as it prevailed here during the past season, was early made on my mind, and I did not employ it in a single instance; but I had an opportunity of observing its effects in several cases to which I was called in consultation. The tendency to sink was generally very great, and the prostration consequent upon free bloodletting was truly alarming, requiring all the powers of medicine to bring about reaction. In former years I have used the lancet with most decided benefit.

*Emetics.*—Many writers condemn the use of emetics in remittent fevers. Dr. Mackintosh cautions young practitioners against their "indiscriminate employment," and remarks "that irritability of the stomach is one of the most frequent and troublesome symptoms, and once excited it is always difficult, in many cases impossible, to restrain it." The sense of weight in the epigastric region, present in all the cases that came under my observation, indicated to me the necessity of emetics in the treatment of the disease. When emetics were administered in the early stage of the disease, they not only relieved the stomach of its contents, but exerted a controlling power which was shown in the modification of every symptom. In a few cases some irritability of the stomach was excited, which was easily controlled by the application of mustard to the epigastrium, followed by cloths saturated with laudanum to the inflamed surface. Some cases required the internal use of morphine, paregoric, or a combination of laudanum and sulphuric ether. Aperients and anti-periodic medicines acted much more promptly and beneficially when preceded by emetics.

*Mercury.*—The symptoms of biliary derangement demanded its employment, but not to the extent to which, in this section of country, it is generally carried. It is contended by some, that the course of a remittent fever may be cut short by bringing on the specific action of mercury; but of the utter fallacy of this doctrine I have had ample and convincing proof. In most of the fatal cases of the past season a severe ptyalism had been induced, and where the result was not so unfavorable the mercurialized patients recovered far more slowly than those who were not treated upon that plan. From the quantity of mercury used, it might be inferred, in the emphatic language of Stokes, that the great mass of our physicians belonged to the "hepatic school of medicine, in which the existence of almost every organ, except the liver, seems to be forgotten, and of which the creed seems to be, that there is but one viscus, the liver; one source of disease, biliary derangement; and one cure, mercury; a creed which, though not defended and enforced by the sword, has cost perhaps as much of human life as others whose history is written in letters of blood." The vast amount of unnecessary and cruel suffering inflicted upon large numbers by the abuse of this medicine, calls loudly for reformation. In many instances the paroxysm of fever returns upon the subsidence of mercurial action. In remittent fever it may be employed in such a way as to avoid its specific effect upon the system, and yet exert a decidedly controlling power over the severity and duration of the disease.

*Sulphate of Quinine.*—The controlling power of this article over the febrile paroxysm is known to all, but there is some difference of opinion as to the proper mode and time of employing it. During the past summer and fall many opportunities were afforded me of observing the effect of quinine when employed previous to the administration of emetics or cathartics, and without reference to the condition of the chylopoietic organs. The high estimation in which Sappington's pills* were held by the community induced many to use them. In the mildest form of simple intermittents they arrested the disease; but in the severer forms of both intermittent and remittent fever, their employment in the early stages was decidedly hurtful; the periodicity of the disease was frequently broken up, but a more malignant and continued fever supervened. When by the use of remedies previously spoken of there was an amelioration of all the symptoms, a reduction of the inflammatory condition of the system, and more distinct remissions, the sulphate of quinine acted as promptly in arresting remittent as intermittent fever. The first dose generally checked, or greatly modified the exacerbation. As to the size of the dose, medical writers differ in opinion. Dr. Stokes, and many physicians in Italy, on the coast of Africa, and in the Southern part of the United States, have expressed a most decided preference for a single large dose once a day. That this practice is successful in many cases, and in many countries, cannot be denied; but that it is always the best practice, and can be indiscriminately employed, may well be doubted. During the

---

* Composed of quinine, liquorice, and gum myrrh.

prevalence of this fever I gave it in various doses and at various intervals—in ten grain doses once a day; in four or five grain doses every three hours, and in half grain doses repeated every half hour. But as a general rule its controlling power was more manifest when given in doses of from two to three grains, repeated at intervals of one hour, for six hours previous to the time when the chill was expected. I am fully persuaded that no one mode of administering it is suited to all cases and climates. There are modifying circumstances in all constitutions, and in all countries; and if these are not carefully noted and weighed, the physician cannot prescribe with any degree of certainty.

May 20, 1846.

